# A Literature Review of the Rare Coexistence of Central Giant Cell Granuloma with Aneurysmal Bone Cyst: A Case Report

**DOI:** 10.22038/ijorl.2021.53844.2838

**Published:** 2021-09

**Authors:** Mahrokh Imanimoghaddam, Samareh Mortazavi, Fereshteh Goudarzi, Nooshin Mohtasham

**Affiliations:** 1 *Department of Oral and Maxillofacial Radiology, School of Dentistry, Mashhad University of Medical Sciences, Mashhad, Iran.*; 2 *Oral and Maxillofacial Diseases Research Center, Faculty of Dentistry, Mashhad University of Medical Sciences, Mashhad, Iran. *

**Keywords:** Aneurysmal bone cyst, Cone-beam computed tomography, Giant cell granuloma, Jaw disease, Maxillary sinus

## Abstract

**Introduction:**

Central giant cell granuloma (CGCG) is a benign bone tumor that occurs more in young females and anterior of the mandible. It can be unilocular or multilocular with wispy-septation, undulating borders, cortical expansion, and perforation. Central giant cell granuloma in association with other benign lesions of the jaws is named hybrid lesion. An aneurysmal bone cyst (ABC) is a rare, rapidly growing benign tumor that is commonly developed in young females and the mandible molar and ramus regions. It is usually a well-defined cyst-like expansile lesion with an internal structure similar to CGC lesions in radiographic features.

**Case Report:**

A 17-year-old girl was referred to the radiology department for panoramic radiography at the end of orthodontic treatment. The complete opacification of the right maxillary sinus, root resorption, and periodontal ligament widening was evident in panoramic radiography. Cone-beam computed tomography revealed a soft-tissue mass and displacement of the lateral nasal wall. The lesion was multilocular with wispy septation and ground glass in some parts. On T2-weighted magnetic resonance imaging, a heterogeneous mass with low to intermediate signals and fluid-fluid levels were observed. The patient underwent surgical curettage, and the histopathological diagnosis was the coexistence of CGCG and ABC.

**Conclusion:**

An unusual view of the coexistence of CGCG and ABC could be a lesion with ground glass pattern calcification. Hybrid lesions with the coexistence of CGCG and ABC are rare, and only six cases are reported in the literature in this regard.

## Introduction

Central giant cell granuloma (CGCG), as an uncommon benign intraosseous lesion with the aggressive ability that accounts for 7% of jaw tumors(1), was described by Jaffe in 1953 for the first time (2). Central giant cell granulomas usually occur in young adults under 30 years of age and in women twice as much as in men (3,4). It has been found that 70% of lesions occur in the mandible anterior to the first molar, and only a few cases have been reported in the maxilla (4,5). 

Radiologically, CGCG can be unilocular or multilocular with wispy-septation, undulating borders, cortical expansion, and perforation. In several case reports, CGCG presented with other benign lesions of the jaws, such as central odontogenic fibroma, ossifying fibroma, fibrous dysplasia, and aneurismal bone cyst, named hybrid lesion.

An aneurysmal bone cyst (ABC) is a rare, rapidly growing benign tumor that affects the mandible more than the maxilla (6). Molar and posterior regions are more often involved than the anterior regions of the jaws. It has been reported that 90% of patients are under 30 years old with a female preference (7). In radiography, it is a well-defined cyst-like expansile lesion with internal structure resemblance to CGC lesions. Both ABC and CGCG are rarely observed in the maxillary sinus (3). This study reported a case of the coexistence of CGCG and ABC in a 17-year-old girl in the maxillary sinus. 

## Case Report

**Fig 1:**
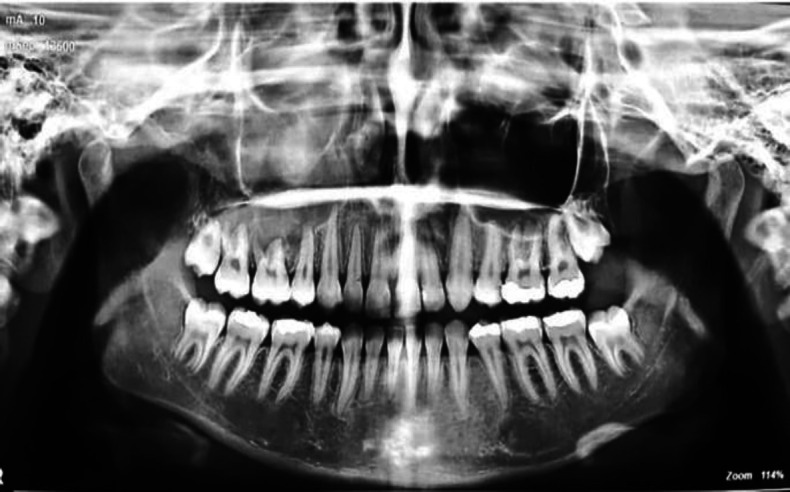
Panoramic radiograph showing opacification in the right maxillary sinus with root resorption of teeth

**Fig 2:**
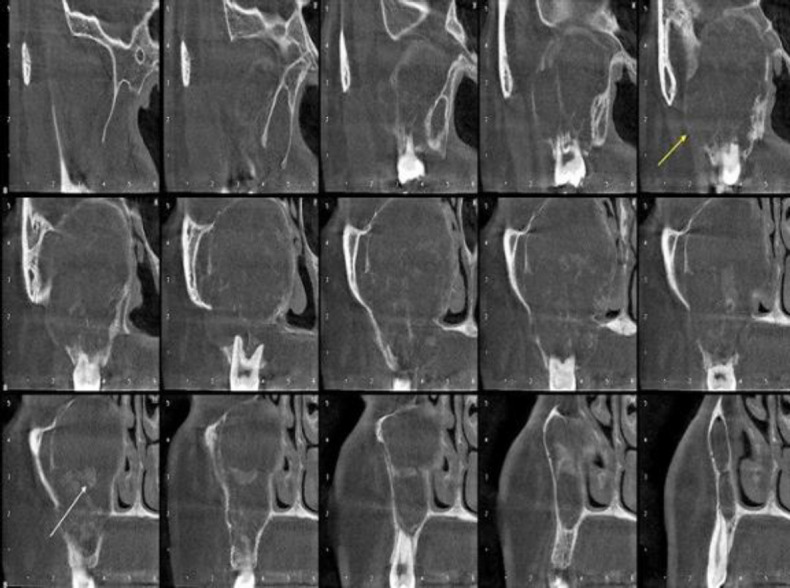
Coronal view of cone-beam computed tomography displayed an expansile lesion with ground glass pattern (short arrow) and erosion of the maxillary sinus’s wall (long arrow)

A 17-year-old girl was referred to the radiology department of Mashhad School of Dentistry, Mashhad, Iran, for panoramic radiography at the end of orthodontic treatment. She did not mention any medical history. On panoramic radiography, the complete opacification of the right maxillary sinus was evident and the inferior border of the sinus was not well apparent in some parts. Root resorption of the second premolar and first molar and periodontal ligament widening with loss of the first molar's lamina dura was also observed (Fig.1). This lesion was accidentally discovered in the patient orthodontic follow-up image. After that, a thorough clinical examination was performed for the patient, and slight asymptomatic swelling was seen in the middle face and hard palate, posterior of the canine tooth. The patient's vision was normal, and she had a history of nasal congestion and antihistamines uptake for a while. For further examination, cone beam computed tomography (CBCT) and magnetic resonance imaging (MRI) were prepared. Cone-beam computed tomography revealed a soft tissue mass completely obliterating the right maxillary antrum with the expansion and thinness of buccal and palatal walls and displacement of the lateral nasal wall. The lesion was multilocular with wispy septation and faint calcifications, which were ground glass in some parts. The lesion was well corticated, and the expansion was uneven (Fig.2).

Based on the radiography features, the aggressive fibro-osseous lesion was introduced as the first diagnosis and we suggested that it should be accompanied by an ABC due to the expansion. Central giant cell granuloma with fibro-osseous lesion was our other differential diagnosis. In coronal sections of MRI T2-weighted, a space-occupying mass was observed in the right maxillary sinus, which had moved the orbital floor and lateral nasal wall.

**Fig 3:**
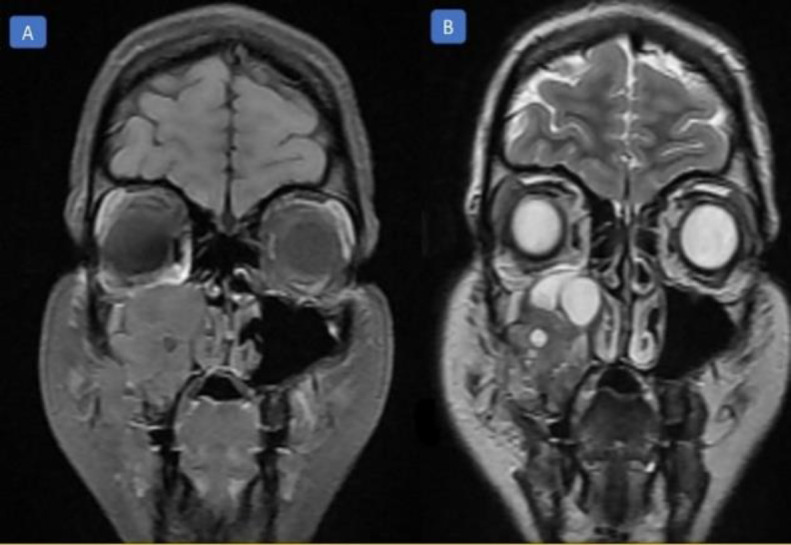
Coronal T1 and T2 weighted magnetic resonance imaging: A) Low-signal soft tissue mass in the right maxillary sinus, B) A non-homogenous lesion with fluid-fluid levels in the right maxillary sinus

**Fig 4:**
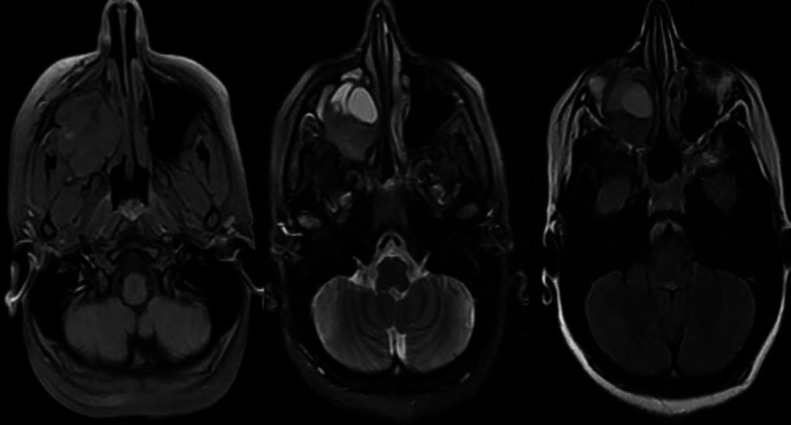
Axial T1, T2, and T2 fluid-attenuated inversion recovery weighted magnetic resonance imaging. There is evidence of abnormal enlarge well-marginated border, and heterogeneous signal soft tissue mass was observed in all sequences

The signal intensity of most of the lesions was intermediate to low, and it was heterogeneous. The lobulated high signal region was evident in superior parts. The mass was extended inferiorly into the alveolar process (Fig.3). In the axial view, it was observed that the lesion was extended to the masticator space from the posterolateral wall of the sinus (Fig.4). 

After general anesthesia, the tumor mass was removed entirely and resected with a 2-cm margin. The central origin of the tumor was the alveolar ridge, palate, and parts of the inferior anterior wall of the maxillary sinus. After removing this area, the rest of the tumor came out of the maxillary sinus as an en bloc. The tumor did not have a connection to other areas, including the superior anterior, medial, posterior walls of the sinus, orbital floor, and pterygoid plate. It was revealed that the floor of the orbit was intact, and the tumor surface was smooth. The excisional biopsy was sent for histopathological diagnosis.

**Fig 5:**
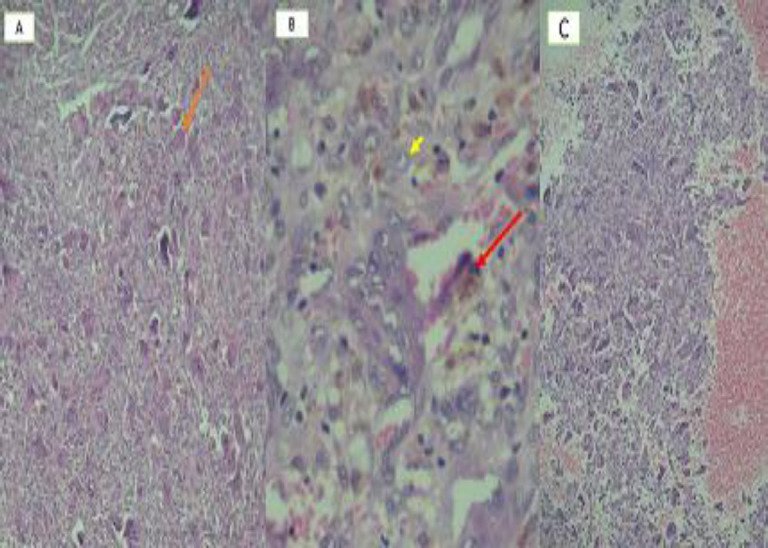
A) Giant cells (arrow; 100x magnification), B) Blood discharge from the red blood cells (arrow); short arrow showing the endothelial cells (400x magnification), and C) Due to present vascular spaces without the endothelial wall, indicating the coexistence with aneurysmal bone cyst lesion (100x magnification)

The microscopic view revealed the proliferation of benign multinucleated giant cells of the foreign body type, endothelial cells with blood vessels' formation, fibroblasts with collagen fibers, and chronic inflammatory infiltration cells (lymphoplasmacytic), red blood cell (RBC), and hemosiderin pigment discharge. Vascular spaces without endothelial walls containing RBC were also observed, implying coexistence with the ABC lesion. The final diagnosis was the coexistence of CGCG and ABC (Fig.5). 

## Discussion

Central giant cell granuloma lesions are divided into two categories based on radiological and clinical findings, namely aggressive and non-aggressive, both of which have the same histopathology (8). The non-aggressive lesions are usually a painless swelling, asymptomatic, and without cortical perforation/root resorption. Nevertheless, the aggressive ones are painful, rapid growing, with bone erosion, cortical perforation, root resorption, extension into the soft tissues, and tendency to be recurrent (1,9). 

The identification of CGCG lesions is essential since not only the lesion itself has some effects but also there is the possibility of malignant transformation, such as fibrosarcoma and osteosarcoma (9). According to the results of a study conducted by Stavropoulos et al., these lesions have been most distinguished on the radiograph as multilocular and well-defined in their systematic review (10,11). Some CGCG types that are considered radiolucent lack internal structure. Other lesions usually have a granular pattern with wispy septa that are considered multilocular (7). The observance of erosion is also common in these cases and is sometimes associated with destruction (5).

The present case was a hybrid CGCG in the maxillary sinus. In many case reports of CGCG in the maxillary sinus, the swelling has been common (Table.1). However, in the present case, the lesion was discovered incidentally because of its expansion into the sinus. In this case, the expansion and thinness of the sinus wall were observed. Table 1 presents some of the case reports (5,9,12,13). 

**Table1:** Comparison of some published cases of central giant cell granuloma in the maxillary sinus

**Case reports **	**Age**	**Gender**	**Symptoms**	**Imaging features**
Balaji (12)	33	Male	Swelling, nasal obstruction, teeth loosening	Well-defined multilocular radiolucency/ expansion/ erosion of the bone
Gulati (5)	19	Male	Swelling	Expansile multilocular radiolucent lesion/ bony erosion and destruction
Saleem (14)	15	Male	Recurrent epistaxis and nasal obstruction	Well-defined/ expansile with a granular bone pattern
Garg (9)	48	Female	Swelling	Soft tissue mass with thinning and destruction
Ramesh (13)	32	Female	Swelling	Diffuse haziness on sinus lytic lesion with a thin ossified rim


The radiographic view in CGCG is not specific and may be similar to other lesions.

Odontogenic myxoma, ameloblastoma, and ABC are suitable differential diagnoses for CGCG when the internal structure is present; the first two ones are distinguished by the type of septa (7). These lesions are usually unifocal, and if multifocal lesions are observed, hyperparathyroidism must be investigated, and bilateral Noonan syndrome and cherubism must be ruled out (1). Aneurysmal bone cyst lesions can be primary, secondary, or coexisting. This lesion has been reported to coexist with the central giant cell lesion, fibrous dysplasia, chondroblastoma, osteoblastoma, fibromyoma, ossifying, and chondromyxoid fibroma (6,15). They can also occur along with some medical conditions, such as unicameral cyst, hemangioma, and histiosarcoma. Although ABC lesions rarely develop malignancies, it is nonetheless essential that they be identified (16). The coexistence of ABC and CGCG lesions in the jaws is rare. However, in the present case, these two lesions occurred in the maxillary sinus, an unusual place for both of them. In their systematic review, Alsufyani et al. collected CGCG and ABC coexistence cases from 1978 to 2019 and reported only four patients (17). In another systematic review, Alsufyani et al. reported the coexistence of these two lesions mostly as corticated, multilocular, and expandable radiolucency (17). In the present study, only a few cases related to these two lesions were found when exploring the Scopus and Google Scholar databases, which are listed in Table 2.

Aurora et al. reported the appearance of these two lesions in one patient in the form of ABC in the maxilla and CGCG in the mandible, which were not considered coexistence (18). Most of the coexistence cases of CGCG and ABC lesions were observed as swelling and expansion, which were the main reasons for patients' referral. This expansion can cause perforation or destruction of the bony cortices. All patients with CGCG coexistence with ABC aged between 2 and 28 years and were predominantly female, and the mandible was the area being more affected. It has been reported that four coexistence cases were radiolucent lesions (17,19-21), and two were multilocular, which was consistent with the results in our patient (3,8). Nevertheless, these results are based on a few cases reported in the literature in six case reports (Table.2). 

**Table 2:** Comparison of the published cases of central giant cell granuloma with an aneurysmal bone cyst

**Case** **reports**	**Age**	**Gender**	**Symptoms**	**Imaging features**	**Location**	**Modality**
Chondolia (3)	28	Male	Painless swelling	Large multilocular radiolucent and radiopaque lesion with root resorption	Posterior of mandible	Panoramic
Pai (8)	2	Male	Swelling	CT: large multiloculated expansive lesion	Coronoid and condylar	CT Panoramic (for postoperative)
Westbury et al. (19)	17	Female	Rapidly painful swelling	Signiﬁcant bony erosion) radiolucent lesion) MRI: a lobulated mass causing erosion and signal of hemorrhage in the ramus of the mandible CT: low-attenuation soft tissue mass with peripheral calciﬁcation	Ramus of mandible	Panoramic CT MRI
Yasuoka et al. (21)	16	Female	Maxillary swelling	Bone defect and well-demarcated radiolucent lesion at the apex of a tooth extending into the maxillary sinus MRI: thickening of the maxillary left sinus Membrane and the air-water interface CT: a cystic lesion and the bone defect	Maxillary sinus	Panoramic CT MRI
Sun et al. (20)	9	Male	Painless facial asymmetry	Unilocular ill-defined radiolucency	Ramus of mandible	-
Padwa (22)	5	Female	Rapidly enlarging swelling	Well-defined unilocular radiolucency CT: expansile lesion of the left mandibular ramus with a thin cortical margin	Molar region to ramus of mandible	Panoramic CT
Present study	17	Female	Asymptomatic	Opacification in the airless sinus Expansion Ground glass pattern on CBCT	Maxillary sinus	Panoramic CT MRI

Due to the presence of osseous radiopaque areas with a ground-glass pattern in the patient's CBCT images, the researchers considered fibro-osseous lesions, such as Central ossifying fibroma, and due to its expansion, coexistence with ABC was supposed in the differential diagnosis. Due to the resemblance of structures to wispy septa, CGCG with fibro-osseous lesions was also another differential diagnosis in our case. According to the results of a systematic review performed by Alsufyani et al., a ground glass pattern is observable only when CGCG coexists with fibro-osseous lesions, which is due to their fibro-osseous portion (17). 

In our study, the case had periodontal ligament widening in some teeth due to orthodontic therapy. This history, along with the lesion's slow course, facilitated differentiating it from such malignancies as osteosarcoma. Based on the MRI images of a study conducted by Westbury et al., a lobulated high signal mass was observed, which was similar to ours (19). Different signals have been mentioned for CGCG and ABC in studies, probably due to differences in the levels of cystic degeneration, haemorrhage or hemosiderin deposits, or osteoid formation. It has been reported that CGCG lesions usually appear as a homogeneous or slightly heterogeneous intermediate signal in T1-weighted, T2-weighted, and short tau inversion recovery (STIR) MRI views. 

On the other hand, ABC lesions typically appear as intermediate-low signals surrounded by a low-signal well-defined rim and fluid-fluid levels in T1-weighted, and as a high signal, fluid-fluid levels in T2-weighted and STIR MRI views (23). 

In the MRI image of the patient in our case report, the diagnostic opinion that helped us diagnose coexistence with ABC was the fluid-fluid levels, which is shown in Figure 3.

Although the fluid-fluid levels are a characteristic feature of ABC, they can also be observed in other lesions, such as giant cell tumors, telangiectatic chondroblastoma, and osteosarcoma (24). 

Histopathology was also used as an adjunct to diagnose such coexistence. The histopathology of CGCG and ABC are similar and only differ in blood collection in variable-sized spaces in ABC (17). 

As mentioned, in giant cell tumors, fluid-fluid levels are also seen on MRI. Nevertheless, giant cell tumors in the jaw bone are rare and more common in long bones. In the histopathological presentation in giant cell tumors, giant cells' distribution is uniform, unlike those in giant cell granuloma (25). On microscopic examination of our case, the distribution of giant cells, along the aneurysmal region, was low. More diffusion was observed in more distant areas, and it was found that the distribution was not uniform (Fig.5(.

Surgical resection was performed to treat the patient. Some studies have described surgical curettage with or without medication (5), while others have used surgical curettage for smaller lesions and en bloc resection for more extensive lesions(7,9). 

**Fig 6:**
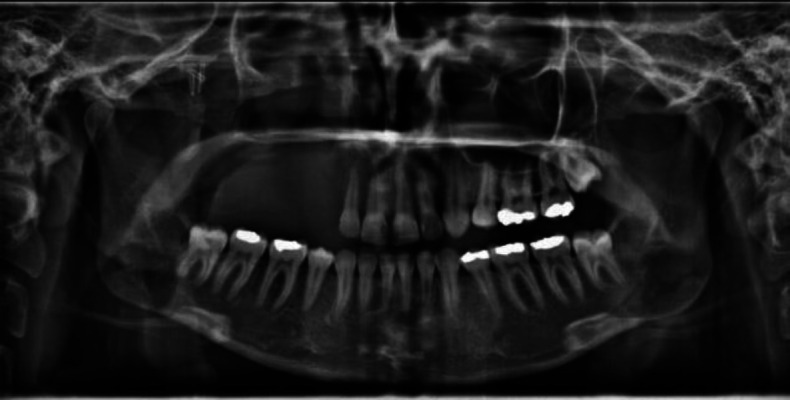
Follow-up image after 6 months

The patient's vision after surgery was normal. No discharge, hematoma, or dehiscence was observed at the site of surgery. In the patient's follow-up after 6 months, no recurrence was observed (Fig.6). 

## Conclusion

The radiographic view of the ground glass pattern for CGCG, which was observed in our case, is rare and similar to the fibro-osseous lesions. The coexistence of CGCG and ABC lesions is unusual and more common in women under 30, with swelling being the most common cause of patient referrals. Since the results of some reports have indicated that both CGCG and ABC lesions can develop malignancies, timely identification and treatment of such lesions are imperative.
